# Breakpoint characterization of a novel large intragenic deletion of *MUTYH *detected in a MAP patient: Case report

**DOI:** 10.1186/1471-2350-12-128

**Published:** 2011-09-30

**Authors:** Giovana T Torrezan, Felipe CC da Silva, Ana CV Krepischi, Érika MM Santos, Fábio de O Ferreira, Benedito M Rossi, Dirce M Carraro

**Affiliations:** 1 International Center of Research and Training (CIPE), A. C. Camargo Hospital, São Paulo, SP, Brazil; 2 National Institute of Science and Technology in Oncogenomics (INCITO), São Paulo, SP, Brazil; 3 Colorectal Tumors Department, A. C. Camargo Hospital, São Paulo, SP, Brazil; 4 Barretos Cancer Hospital-Pio XII Foundation, Barretos, São Paulo, Brazil

## Abstract

**Background:**

*MUTYH*-associated polyposis (MAP) is a recessive, hereditary, colorectal cancer-predisposing syndrome caused by biallelic mutations in the *MUTYH *gene. Most *MUTYH *pathogenic variants are missense mutations, and until recently no gross genomic deletions had been described.

**Case Presentation:**

We have identified a large deletion in the *MUTYH *gene: a > 4.2 kb deletion encompassing exons 4-16. This is the second description of this rearrangement, which has been recently described as the first large deletion in this gene. The clinically suspected MAP patient was homozygous for this mutation and presented with no amplification products for 14 exons of *MUTYH *on initial screening. Deletion breakpoints were refined to base pair level through array comparative genomic hybridization (aCGH) analysis followed by sequencing. The identified breakpoints were located within intron 3 and 146 bp downstream of the 3' end of the gene, with the presence of an *Alu*Jr element adjacent to the distal breakpoint. The presence of a 2 bp insertion at the junction suggests the involvement of the non-homologous end joining (NHEJ) repair mechanism, possibly facilitated by rearrangement-promoting elements. Examination of the *MUTYH *locus revealed a high *Alu *density that may make this region prone to rearrangements.

**Conclusion:**

Large deletions are a possible mechanism for loss of function of the *MUTYH *gene, and investigation of such mutations may be important in identifying causative mutations in MAP patients.

## Background

*MUTYH*-associated polyposis (MAP) (MIM#608456), a recessive inherited syndrome characterized by colorectal adenomatous polyposis and a high risk of colorectal cancer, is a disorder caused by biallelic pathogenic germline variants in the *human mutY homologue *(*MUTYH*) gene [1]. *MUTYH *spans 11.2 kb on chromosome 1p34.1 [2] and encodes a DNA glycosylase that plays a key role in base excision repair (BER) of 8-oxoG:A mismatches by removing the mismatched adenine [3]. The oxidation product 8-oxoG is the most stable product of oxidative DNA damage [4], which can lead to G:C to T:A transversions if not repaired^5^. Tumorigenesis in MAP patients is thought to be initiated by somatic G:C →T:A transversions in *KRAS *and/or *APC *[5].

The clinical manifestations of MAP resemble familial adenomatous polyposis (FAP; MIM#175100), which is caused by autosomal dominantly inherited pathogenic variants in the *APC *gene. Most biallelic *MUTYH *mutation carriers have between ten and several hundred polyps, usually with later onset compared to FAP patients [1,6]. Also, a number of MAP patients with CRC and no polyps have been reported [5]. Mutations in *MUTYH *account for approximately 40% of patients with 10-100 colorectal adenomas (attenuated FAP patients) and positive familial history, a proportion slightly higher than that of *APC *mutations in these patients (30%) [7].

Nearly 300 different sequence variants have been identified so far in this gene (*MUTYH *Leiden Open Variation Database; http://chromium.liacs.nl/LOVD2/colon_cancer/home.php?select_db=MUTYH). In contrast to pathogenic *APC *variants, which mostly result in a truncated or absent protein, most pathogenic *MUTYH *variants are missense variants and only a minority consists of splice site and truncating variants [2] The two hotspot mutations p.Tyr179Cys (exon 7) and p.Gly396Asp (exon 13) are prevalent in populations of European origin, probably due to a founder effect, and account for approximately 80% of all reported mutant alleles [5,8]. In the Brazilian population, a recently published paper on patients with clinical phenotypes of MAP, FAP and Lynch syndrome identified 4/60 (6.6%) patients with these two hotspot mutations in a biallelic state [9]. Until recently, no gross genomic deletions or duplications had been described in this gene in any population.

Here, we report a case study in which we have characterized a large *MUTYH *deletion in a MAP patient. During the composition of this manuscript, an independent study performed by a French group identified this same rearrangement in one of their polyposis patients [10]. We have refined the breakpoint of this > 4.2 kb deletion to the base pair level. Based on the analysis of the sequences at breakpoints, we suggest a possible mechanism of origin for this alteration. Presence of this deletion was also analyzed in familial colorectal patients and a control group.

### Case Presentation

**Figure 1:**
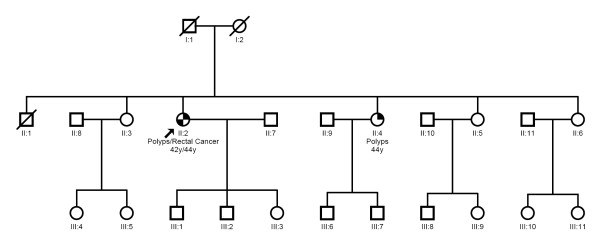
**Family tree of patient FAP15**. The proband is indicated by the arrow. The proband's age at onset of the polyposis (42 years) and of the rectal cancer (44 years) are indicated. The patient presented unaffected deceased parents, four unscreened (without colonoscopy) siblings and one affected sister. The affected sister presented with polyposis at the age of 44 years. The Caucasian patient stated having Portuguese great-grandparents and no report of inbreeding in her family.

A Brazilian female patient (FAP15) from the Hereditary Colorectal Cancer Registry of Hospital AC Camargo (São Paulo, Brazil), who was clinically suspected for MAP, was screened for mutations in the *MUTYH *gene by direct sequencing. The age at onset of the attenuated polyposis (approximately 40 polyps) was 42 years and of the rectal cancer was 44 years. No extracolonic manifestations were observed in this patient. Family history was accessed through personal report. The patient stated that she had unaffected deceased parents, four unscreened siblings (without colonoscopy) and one affected sister from whom biological material was unavailable due to residence distance (Figure 1). The affected sister presented with attenuated polyposis at the age of 44 years. The proband had a Caucasian ancestry, since her great-grandparents were Portuguese.

In addition, the presence of this *MUTYH *deletion was screened through PCR in other 183 Brazilian individuals: three *APC/MUTYH*-mutation negative and 18 *APC/MUTYH*-mutation positive polyposis patients (12 *APC *mutation carriers, 5 *MUTYH *biallelic and one *MUTYH *monoallelic mutation carriers); 51 clinically suspected Lynch syndrome patients (fulfilling Amsterdan II or Bethesda criteria), who were non-carriers of germline mutations in the mismatch repair (MMR) genes *MLH1*, *MSH2*, *MSH6 *and *PMS2*; and 111 healthy controls. Written informed consent was obtained from all patients and controls. This study was approved by the ethics committee of A. C. Camargo Hospital (approval number: 1169/08-B).

### Identification and characterization of the deletion

**Figure 2:**
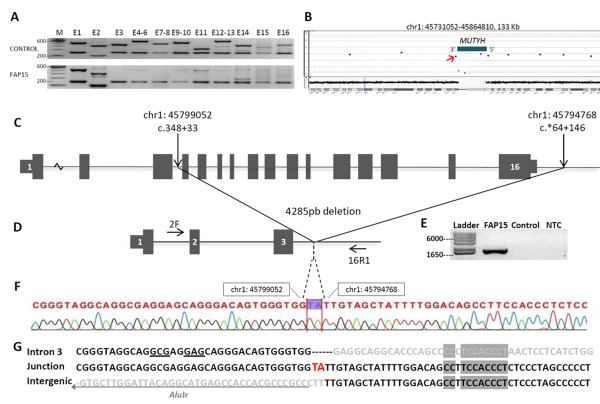
**Homozygous deletion and characterization of the breakpoint in patient FAP15**. **A: **Agarose gel of multiplex PCR performed for *MUTYH *exons 1 to 16 (E1-E16) and control genes *GAPDH *or *HPRT1*. The patient (FAP15) exhibited absence of amplification for *MUTYH *exons 3 to 16, whereas a healthy individual (Control) presented all expected fragments. **B: **The blue line on the profile of chromosome 1 (bottom panel) indicates the mapping at 1p34.1 of the two intragenic probes with log_2 _ratio values compatible with a homozygous deletion (green circles, upper panel); probes flanking this deletion (at 5' of *MUTYH*, and downstream of its 3' end) exhibited normal values. The red arrow indicates the position of probe A_14_P118536 where novel reverse primers were designed. **C: **Schematic drawing of the normal gene with genomic and cDNA (NM_001128425.1) positions of the breakpoints. **D: **Schematic drawing of the deleted gene and primer locations (2F primer: chr1: 45800257-45800276; 16R1 primer: chr1: 45794388-45794407). **E: **Agarose gel showing the amplification of the deletion junction in the patient and amplicon absence in the control DNA pool. **F: **Partial chromatogram of the junction sequencing showing in highlight the two inserted nucleotides (TA). **G: **Nucleotide sequence around the deletion breakpoints and the deletion junction. Red larger nucleotides are the non-template insertion (filler DNA). Light gray nucleotides indicate the deleted sequence. Shaded nucleotides represent shared sequences between the breakpoints. Rearrangement-promoting elements GAG/GCS* are shown underlined. The gray arrow beneath the intergenic deleted sequence shows the location of the *Alu*Jr element. *S denotes the ambiguity code symbol for G/C. Coordinates at chromosome 1 are given according UCSC Feb. 2009 (GRCh37/hg19) assembly.

We detected a homozygous deletion encompassing several exons of the *MUTYH *gene in this MAP patient. Genomic DNA was obtained from blood using the Puregene Genomic DNA Isolation Kit (Gentra Systems, USA) according to manufacturer's instructions. Eleven primer pairs were designed to amplify all 16 exons of *MUTYH *gene (NM_001128425.1), including intron-exon boundaries. Primers used for these analyses and PCR conditions are available upon request. Unexpected results were observed when performing standard PCR for *MUTYH *mutation screening on this patient; exons 3-16 could not be amplified (data not shown). As a positive control for the reaction, multiplex PCR in the presence of primers for control genes (*GAPDH *intron 7; *HPRT1 *exon 3) was performed for each *MUTYH *exon and confirmed the absence of genomic template for this gene. The results of multiplex PCR for the patient and a control DNA are shown in Figure 2A.

With the purpose of elucidating the extent of the deletion, comparative genomic hybridization based on microarrays (aCGH) was performed. Briefly, samples were labeled with Cy3- and Cy5-dCTPs by random priming. Experiments were performed in duplicate using a 180 K whole-genome platform (OGT Technologies). Purification, hybridization, and washing were performed as recommended by the manufacturer. Data extraction was conducted using the Feature Extraction software (Agilent Technologies). We applied the Genomic Workbench software (Agilent Technologies) for identifying the constitutive genomic imbalances using the statistical algorithm ADM-2, with a sensitivity threshold of 6.7, a threshold log_2 _ratio of 0.4 or 1.1 for duplication or high copy number gain, and -0.4 and -1.1 for deletion and homozygous loss, respectively.

Data analysis detected two sequences mapped within the *MUTYH *gene that exhibited log_2 _ratio values compatible with a homozygous deletion (oligoprobes A_14_P107667, mapped at chr1:45794902-45794961, and A_14_P122926, at chr1:45797594-45797640; coordinates given according to UCSC Feb. 2009 (GRCh37/hg19) assembly) (Figure 2B). Oligoprobes flanking the deletion at the 5' end of *MUTYH *gene sequence and downstream of the 3' end had log_2 _ratios values consistent with two copies (A_14_P122926-chr1:45804348-45804405, and A_14_P118536-chr1:45793780-45793830) (Figure 2B). Using an exon 2 forward primer (5'-GGTGGATGAGAGGGAGATAG-3') and reverse primers designed on the position of the A_14_P118536 undeleted probe and 600 bp upstream (primers 16R2: 5'-CTTCAGGTCTTCACCAAGTCC-3' and 16R1: 5'-CTTCTCCTGTGCTTCCTCTC-3', respectively), we successfully amplified the junction fragments for this deletion. After confirming that the primer 16R1 was located at an undeleted region and as it was closest to the breakpoint, further analyses were performed only with this primer. The PCR reaction of the control DNA pool failed to amplify any detectable PCR product (theoretically 5889 bp in length), whereas PCR products of ≈ 1600 bp were detected using genomic DNA from the patient (Figure 2E). Briefly, the PCR conditions consisted of 25 ng of genomic DNA, 0.3 μmol of each primer and 1X Platinum^® ^PCR SuperMix High Fidelity (Invitrogen) in a 20 uL reaction. Cycling conditions entailed a preincubation at 95°C for 2 minutes followed by 35 cycles of denaturation at 95°C for 20 seconds, annealing at 62°C and extension at 72°C for 2 minutes. Direct sequencing of the junction fragments identified the exact breakpoints (genomic position: chr1:g.45,794,768_45,799,052del4285insTA, cDNA position: c.348+33_*64+146del4285insTA), the precise size of the deletion interval (4,285 kb) and the affected exons (4-16) (Figure 2C and 2D). Genomic positions were confirmed using MUTALYZER 2.0 software. Previously, exon 3 appeared to be deleted in the PCR analysis because the breakpoint is located upstream of the reverse primer site. No region of the adjacent *HPDL *gene is deleted downstream of the 3' end of *MUTYH*.

As the patient investigated in this study presented a homozygous mutation and reported no inbreeding in her family, we examined the presence of this deletion among 111 healthy controls, 21 polyposis patients and 51 patients with familial CRC negative for mutations on MMR genes. The junction fragment could not be amplified from the DNA of any other patient or control (data not shown). One possible explanation for the homozygous occurrence of the deletion in this patient, without consanguineous parents, is the inheritance of two alleles of a founder mutation in the population. The proband's great-grandparents were Portuguese, therefore she had Caucasian ancestry. In this sense, very recently this same deletion was found in a European MAP patient in compound heterozygosity with the common *MUTYH *Caucasian mutation p.Gly396Asp [10]. The description of the same rearrangement in patients from different geographic regions is a very exciting discovery and additional studies to uncover the origin and frequency of this mutation would be of great value. Another potential explanation for the homozygosity is the occurrence of isodisomy, a type of uniparental disomy that results in two identical segments from one parental homologue and can occur due to a recombination event in the zygote,, though the existence of an affected sibling does not support this hypothesis.

**Figure 3:**
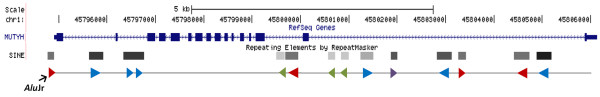
**Short interspersed nucleotide element (SINE) content of the *MUTYH *genomic region (chr1:45794343-45806142) (data from UCSC database and RepeatMasker)**. A region of 11.8 kb is shown, extending 571 bp downstream of the *MUTYH *gene. Vertical bars in the genomic sequence indicate exons. The direction of the arrowheads denotes the orientation of the SINE repeat elements and each family is represented by a different color (Red: *Alu*J, Blue: *Alu*S, Green: MIR, Purple: FLAM_C). The black arrow indicates the *Alu*Jr element involved in the distal breakpoint; no repetitive element is present on the proximal breakpoint.

Analysis of the junction sequence revealed several important features, including an insertion of 2 bp of non-template DNA (Figure 2F), several recombination motifs such as GCG and GAS [11] and the presence of an *Alu*Jr element located 2 bases upstream of the distal breakpoint (Figure 2G). No repetitive element is present at the upstream breakpoint. Examination of the *MUTYH *locus using the Repeat-Masker software http://www.repeatmasker.org/ revealed an *Alu *density of 22.2% for an 11,800 kb region (chr1:45,794,343-45,806,142), which includes the entire gene (11,229 bp), and 571 bp downstream, which encompasses the distal breakpoint characterized in this study (Figure 3).

This study is particularly important from both a clinical and research perspective because we have identified the first large intragenic deletion in *MUTYH*: a 4.285 kb homozygous deletion in a MAP patient (c.[348+33_*64+146del4285insTA];[348+33_*64+146del4285insTA]). This mutation was submitted to the *MUTYH *LOVD Mutation Database and has been publicly available since May 12th: http://chromium.liacs.nl/LOVD2/colon_cancer/variants.php?select_db=MUTYH&action=view&view=1012041%2C0002450%2C10. The patient presented a homozygous deletion encompassing exons 4 to 16 detected and mapped through PCR, aCGH and sequencing. Breakpoints were sequenced and analysis of the junction sequence allowed us to postulate that classical nonhomologous end joining (NHEJ) is the most likely mechanism responsible for the deletion, as a 2-bp non-template insertion sequence is observed at the breakpoint junction [12,13]. This repair process involves the double strand breakage of DNA followed by end joining in the absence of extensive sequence homology, and is associated with small insertions at the junction sites [14] and with very short stretches of sequence identity (a few bp) between the two ends of the breakpoint junctions [15]. Analysis of breakpoints surrounding sequences revealed the presence of an *Alu*Jr adjacent to the distal breakpoint and the existence of recombination/deletion promoting motifs [11] such as GAG and GCS trinucleotides. These motifs are known DNA polymerase pause-site core elements [16] and might have facilitated deletion.

*In silico *analysis of the *Alu *content in the *MUTYH *gene region revealed a high ratio of *Alu *sequences per kb, with *Alu *elements occurring once in every 1.8 kb (22.2%) when compared with the average of the human genome where *Alu *elements account for about 10% and occur once every 3 kb [17]. A recent study of more than 20 genes found evidence that a high content of transposable elements causes increased frequency of gene disruption by gross deletions in human disease [18]. Additionally, the local enrichment of *Alu*s has been observed in regions with recurrent *Alu*-mediated rearrangements, e.g., the *VHL *locus in von Hippel-Lindau disease patients [19], and in genes implicated in colorectal cancer-predisposing disorders such as Lynch and Peutz-Jeghers syndromes (*EPCAM-MSH2 *[20] and *STK11 *[21], respectively). However, not every *Alu*-rich gene is prone to this type of deletion formation. Examples include the thymidine kinase or b-tubulin genes [20,22]. In the *MUTYH *gene, a recent study has shown that a polymorphic *Alu*Y insertion in intron 15 when in a homozygous state, leads to a 2.15 fold increase in the levels of 8-oxoG in leukocyte DNA and is associated with type 2 diabetes mellitus [23]; however, this insertion has not yet been studied in the context of colorectal cancer.

## Conclusions

Currently, there is no information on the percentage of defects in the *MUTYH *gene caused by deletions/duplications of complete exons, and screening for this type of mutation is not a common practice as it is with the *APC *gene. Our finding highlights the importance of performing screening methods, such as qPCR, MLPA or long-range PCR, for large *MUTYH *deletions in polyposis patients. Furthermore, it remains to be addressed whether the high density of *Alu *elements in this gene can cause an elevated frequency of *Alu*-mediated deletions and rearrangements. Nevertheless, the evidence shown here suggests that deletions could represent an important mechanism of loss of function in the *MUTYH *gene.

## Consent

Written informed consent was obtained from all patients and controls for publication of this case report and any accompanying images. Copies of the written consents are available for review by the Editor-in-Chief of this journal.

## Competing interests

The authors declare that they have no competing interests.

## Authors' contributions

GTT designed and performed the molecular genetic studies and drafted the manuscript. FCCS participated in the experimental design and revised the manuscript critically. ACVK carried out the aCGH analysis and revised the manuscript critically. EMMS participated in the clinical data acquirement and analysis. FOF conceived the study, and participated in its coordination. BMR conceived of the study, and participated in its design. DMC participated in its experimental design and coordination and helped to draft the manuscript. All authors read and approved the final manuscript.

## Pre-publication history

The pre-publication history for this paper can be accessed here:

http://www.biomedcentral.com/1471-2350/12/128/prepub
